# A4 DNA HYPOMETHYLATION INHIBITS COLITIS-ASSOCIATED CANCER

**DOI:** 10.1093/jcag/gwad061.004

**Published:** 2024-02-14

**Authors:** F Larsen, H Good, A Shin, M Derouet, L Zhang, C Castellani, S Asfaha

**Affiliations:** Western University, London, ON, Canada; Western University, London, ON, Canada; Western University, London, ON, Canada; Western University, London, ON, Canada; Western University, London, ON, Canada; Western University, London, ON, Canada; Western University, London, ON, Canada

## Abstract

**Background:**

Colorectal cancer is the second leading cause of cancer death in Canada. A major risk factor for the development of colorectal cancer is chronic inflammation leading to colitis-associated cancer (CAC). We previously described a CAC mouse model in which tumors arise from DCLK1+ cells following loss of the tumor suppressor adenomatous polyposis coli (APC) and induction of colitis. Interestingly, both colitis and CAC are associated with DNA methylation changes that lead to altered gene expression. Moreover, inhibition of DNA methylation has recently been shown to lead to a viral mimicry response. The effects of DNA methylation on colonic tumorigenesis, however, is not known.

**Aims:**

Thus, we aim to investigate whether inhibition of DNA methylation leads to a viral mimicry response that inhibits colitis-associated tumorigenesis.

**Methods:**

We first examined the effects of DNA hypomethylation on CAC, by crossing Dclk1-CreERT2/Apc^f/f^ mice to Dnmt1^f/f^ mice which allowed for knock-out of the DNA methyltransferase enzyme DNMT1 in DCLK1+ cells. Dclk1/Apc^f/f^/Dnmt1^f/f^ and Dclk1/Apc^f/f^ (control group) mice were administered three doses of tamoxifen followed by 2.5% dextran sodium sulfate (DSS) for five days to induce colitis. Fourteen weeks later, we assessed colonic tumor number and size. In a separate cohort of Dclk1/Apc^f/f^ mice treated with DSS, we compared colonic tumor number in mice treated with six doses of 5-AZA-2’-deoxycytidine (5-AZA) versus vehicle. The effects of 5-AZA and DSS on DNA methylation were assessed using the Infinium Mouse Methylation BeadChip Array on colonic epithelial cells isolated from mice treated with vehicle versus 5-AZA in the presence or absence of colitis. RNA expression of viral transposable elements and type I interferon response related genes were measured as a readout of a viral mimicry response. Lastly, we investigated the mechanism by which DNA hypomethylation affects tumorigenesis by crossing Dclk1/Apc^f/f^/Dnmt1^f/f^ mice to MAVS knockout mice and examining tumor number following tamoxifen and DSS administration as above.

**Results:**

Deletion of DNMT1 in DCLK1+ cells or treatment with 5-AZA significantly decreased colonic tumor number and size. Treatment with 5-AZA or DNMT1 loss led to DNA hypomethylation and subsequent upregulation of endogenous retroviral expression. Both 5-AZA treatment and DNMT1 knockout in DCLK1+ cells led to increased expression of type I interferon response genes. Knockout of the type I interferon response related gene, MAVS, reversed the anti-tumor effect observed with DNMT1 loss.

**Conclusions:**

Our findings demonstrate that DNA hypomethylation by 5-AZA or loss of DNMT1 reduces colitis-associated tumorigenesis through activation of a viral mimicry response.

**Funding Agencies:**

CAG, CIHRCancer Research Society, Canada Foundation for Innovation, Lawson Health Research Institute

